# Changes in the transcriptional profile in response to overexpression of the osteopontin-c splice isoform in ovarian (OvCar-3) and prostate (PC-3) cancer cell lines

**DOI:** 10.1186/1471-2407-14-433

**Published:** 2014-06-13

**Authors:** Tatiana M Tilli, Akeila Bellahcène, Vincent Castronovo, Etel R P Gimba

**Affiliations:** 1 Coordenação de Pesquisa, Programa de Carcinogênese Molecular, Instituto Nacional de Câncer (INCa)/Programa de Pós Graduação Stricto Sensu em Oncologia do INCa, Rio de Janeiro, RJ, Brazil; 2 Metastasis Research Laboratory, Grappe Disciplinaire de Génoprotéomique Appliquée (GIGA) Cancer, Liège University, Liège, Belgium; 3 Departamento Interdisciplinar (RIR), Instituto de Humanidades e Sáude, Universidade Federal Fluminense, Rua Recife, s/n –Bairro Bela Vista, Rio das Ostras, RJ, Brazil

**Keywords:** Osteopontin, Splicing isoform, Gene expression, PCR array, Angiogenesis

## Abstract

**Background:**

Especially in human tumor cells, the osteopontin (OPN) primary transcript is subject to alternative splicing, generating three isoforms termed OPNa, OPNb and OPNc. We previously demonstrated that the OPNc splice variant activates several aspects of the progression of ovarian and prostate cancers. The goal of the present study was to develop cell line models to determine the impact of OPNc overexpression on main cancer signaling pathways and thus obtain insights into the mechanisms of OPNc pro-tumorigenic roles.

**Methods:**

Human ovarian and prostate cancer cell lines, OvCar-3 and PC-3 cells, respectively, were stably transfected to overexpress OPNc. Transcriptomic profiling was performed on these cells and compared to controls, to identify OPNc overexpression-dependent changes in gene expression levels and pathways by qRT-PCR analyses.

**Results:**

Among 84 genes tested by using a multiplex real-time PCR Cancer Pathway Array approach, 34 and 16, respectively, were differentially expressed between OvCar-3 and PC-3 OPNc-overexpressing cells in relation to control clones. Differentially expressed genes are included in all main hallmarks of cancer, and several interacting proteins have been identified using an interactome network analysis. Based on marked up-regulation of *Vegfa* transcript in response to OPNc overexpression, we partially validated the array data by demonstrating that conditioned medium (CM) secreted from OvCar-3 and PC-3 OPNc-overexpressing cells significantly induced endothelial cell adhesion, proliferation and migration, compared to CM secreted from control cells.

**Conclusions:**

Overall, the present study elucidated transcriptional changes of OvCar-3 and PC-3 cancer cell lines in response to OPNc overexpression, which provides an assessment for predicting the molecular mechanisms by which this splice variant promotes tumor progression features.

## Background

Osteopontin (OPN) is a secreted, integrin-binding phosphoprotein that has been clinically and functionally associated with cancer and is overexpressed in different tumor types [1,2]. Several studies on ovarian and prostate carcinomas have demonstrated increased OPN expression, which has been associated with advanced tumor stage, poor patient prognosis and metastasis formation [3,4]. OPN functional diversity has been associated with several post-translational modifications that cause OPN proteins to differentially bind to integrin and CD44 receptors [2]. Another mechanism underlying the functional diversity of OPN is the existence of splice variants (OPNa, OPNb and OPNc). OPNa is the full-length isoform, while OPNb and OPNc lack exons 5 and 4, respectively [5].

We recently published the first reports about OPN splicing isoforms (OPN-SI) in ovarian and prostate carcinomas, by demonstrating the expression patterns and functional roles of each OPN-SI in these tumor models [6-8]. We showed that OPNc is specifically expressed in ovarian tumors, compared to benign and non-tumoral ovarian samples [6]. We also observed that among the three OPN-SI, OPNc is the most up-regulated splice variant in prostate cancer samples, and outperformed the remaining isoforms and prostate-specific antigen (PSA) serum levels in the accuracy of prostate-cancer diagnosis [7]. Based on these data, we addressed the function of each OPN-SI in ovarian and prostate carcinomas by examining the effect of their ectopic overexpression in OvCar-3 and PC-3 cells, respectively. OPNc overexpression increased OvCar-3 and PC-3 cell growth, and sustained proliferative survival, migration, invasion, anchorage-independence and tumor formation *in vivo*, suggesting a possible role for OPNc in the progression of ovarian and prostate carcinomas. Additionally, we demonstrated that these tumor-promoting effects were mediated mainly through activation of the Phosphatidylinositol-3 Kinase (PI3K)/Akt signaling pathway. In the prostate cancer cell line model, we demonstrated that OPNb also stimulated all these tumor-progression features, although to a lesser extent than OPNc [6,8].

The role of each OPN-SI is tumor-specific, although the mechanisms controlling these patterns are currently unknown [5]. The putative signaling pathways mediating full-length OPN roles have been investigated in breast cancer and hepatocellular carcinomas [5,9]. However, none of these reports is related to OPN splice variants and their transcription-related patterns. Although we have described some of the OPNc functional roles in ovarian and prostate carcinoma progression, the molecular mechanisms mediating these pro-tumorigenic features have not been characterized. A description of the genes and signaling pathways modulating the roles of OPNc in these tumor models might improve understanding of its tumor-specific properties. In addition, this characterization could indicate additional roles of OPNc in different aspects of tumor progression. In the current report, we used a multiplex real-time PCR Cancer Array comprising genes involved in the main hallmarks of cancer, as an experimental approach to identify signaling pathways that are modulated by OPNc in ovarian and prostate carcinoma-overexpressing cells, in comparison to empty vector-transfected cells. Our data indicated that OPNc-overexpressing cells cause specific transcriptional patterns in ovarian and prostate carcinoma cell line tumor models, which are correlated with key cancer pathways. We believe that this is the first study focusing on OPNc downstream molecules in both types of tumors in response to the overexpression of this tumorigenic splice variant. Considering the marked up-regulation of the *Vegfa* transcript in response to OPNc overexpression in both OvCar-3 and PC-3 cells, and also previous data from our group demonstrating that conditioned medium (CM) secreted from cells overexpressing OPNc (OPNc-CM) is able to stimulate most OPNc tumor-causing features [6,8], we used this CM to further validate part of these array data. We functionally demonstrated that OPNc-CM secreted by OvCar-3 and PC-3 cells overexpressing OPNc stimulates proliferation, migration and adhesion of endothelial cells, as evidenced by the PCR array transcriptomic profile.

## Methods

### Cell culture, OPN plasmids and transfection

As a model to examine the signaling pathways modulated by OPNc overexpression in ovarian and prostate carcinomas, we used OvCar-3 and PC-3 cell lines, which were provided by ATCC. All cell lines were cultured in medium supplemented with 20% (OvCar-3) or 10% (PC-3) fetal bovine serum (FBS), 100 IU/mL penicillin and 100 mg/mL streptomycin in a humidified environment containing 5% CO_2_ at 37°C. The OPNc expression plasmids were donated by Dr. George Weber (Univ. of Cincinnati, USA). The open reading frame of OPNc was cloned into the pCR3.1 mammalian expression vector as previously described [6,8]. Transfections were performed using Lipofectamine™ 2000 (Invitrogen, CA). OvCar-3 and PC-3 stably transfected cells contain high levels of protein and transcript of OPNc isoform in relation to their endogenous levels in empty vector-transfected cells (Additional file 1). Cells transfected with empty vector (EV) were used as a negative control in these assays. HUVEC cells were isolated and cultivated as described previously [10]. This work has been approved by the Research Ethics Committee from National Institute of Cancer (INCA).

### Human cancer pathway finder PCR array

The Human Cancer Pathway Finder SuperArray (PAHS-033A; Qiagen) was used to determine changes in the specific genes encoding proteins related to the main hallmarks of cancer in response to OPNc overexpression. The assay design criteria ensure that each qPCR reaction will generate single, gene-specific amplicons and prevent the co-amplification of non-specific products. The qPCR Assays used in these PCR Arrays were optimized to work under standard conditions, enabling a large number of genes to be assayed simultaneously. Similar qPCR efficiencies, greater than 90%, have been used for accurate comparison among genes.

We analyzed mRNA levels of 84 genes related to cell cycle control, apoptosis and cell senescence, signal transduction molecules and transcription factors, adhesion, angiogenesis, invasion and metastasis; and also 5 housekeeping genes and genomic DNA contamination controls. The PCR plates were run using the CFX96 Real-Time System cycler (BioRad, Hercules, CA), following a superarray two-step cycling PCR protocol, in which each plate ran one cycle for 10 min at 95°C, as well as 40 cycles of 95°C for 15 sec and 60°C for 1 min. Based on described high reproducibility of this PCR array system, we used technical triplicates for each tested and control cDNA samples. After the super array protocol was run for each plate, RT-PCR data were analyzed using the website: http://www.SABiosciences.com/pcrarraydataanalysis.php, in order to compare gene expression of OPNc-overexpressing cells and empty vector transfected cells. Total RNA quality control, cDNA synthesis and the quantitative real-time RT-PCR (qRT-PCR) array were performed as recommended by the manufacturer (Qiagen). Data for gene expression were analyzed using standard Excel-based PCR Array Data Analysis software provided by the manufacturer (Qiagen). Fold-changes in gene expression were calculated using the ΔΔCT method, and five stably expressed housekeeping genes (β2 microglobulin, hypoxanthine phosphoribosyltransferase 1, ribosomal protein L13a, GAPDH and β-actin) were used to normalize the level of expression. Array data have been deposited at GEO repository and can be accessed by the GSE57904 reference number at: http://www.ncbi.nlm.nih.gov/geo/query/acc.cgi?acc=GSE57904. The statistical analysis was performed to compare the gene expression values for the OPNc-overexpressing cells and those transfected with empty vector. P < 0.05 was considered statistically significant. Only genes showing a 1.5-fold or greater change were considered for further analysis.

### Transcriptome–interactome analysis

We and others have used a system biology approach to analyze protein–protein interaction (PPI) networks [11]. In order to prepare a protein interaction map of genes that are differentially expressed in OC and PCa overexpressing OPNc in relation to control cells, PPI networks were examined through literature searches (PubMed), PPI databases (Human Protein Reference Database, HPRD), and functional protein association networks (STRING, OPHID) (http://string.embl.de/newstring_cgi/show_input_page.pl). To identify which genes in the databases corresponded to genes listed in the Human Cancer Pathway Finder PCR Array data, we used either the gene symbol or the SwissProt entry name shown in the protein databases.

### Preparation of conditioned medium

In order to prepare the CM secreted from cell clones, the cell number was normalized by plating OvCar-3 and PC-3 at the same cell density (5×10^5^ cells) for each individual OPNc and EV-overexpressing cell clone. After reaching 80% cell confluence, cells were washed twice with phosphate-buffered saline and cultured with RPMI in serum-free conditions for 48 h. Collected CM was clarified by centrifuging at 1500 rpm for 5 min. All functional assays were performed using freshly prepared CM. Total protein concentration of this CM was measured using the BCA assay kit (BioRad) with bovine serum albumin as a standard, according to the manufacturer’s instructions. In each CM sample, we used 150 μg of total protein extract. CM produced by OPNc-overexpressing cells or those transfected with EV controls, which were termed OPNc-CM and EV-CM, respectively, were used for HUVEC endothelial adhesion, proliferation and migration assays, as described below. OPNc overexpression was analyzed by qRT-PCR and immunoblot.

### HUVEC adhesion, migration and proliferation assays

For adhesion assays, 96-well bacteriological plates (Greiner Bio-One) were coated with OPNc-CM and EV-CM, which were used as coating substrates for HUVEC adhesion. HUVEC cells were seeded at a density of 2x10^4^ cells and incubated at 37°C for 2 h in either the OPNc-CM or EV-CM pre-coated wells. Attached cells were stained with crystal violet, and the cell-incorporated dye was quantified by measuring absorbance at 550 nm with a SPECTRAmax GEMINI-XS, using SoftMax Pro software Version 3.1.1.

HUVEC cell-migration assays were evaluated by *in vitro* wound-closure assay, as described by others [12]. Wild-type HUVEC cells were grown in six-well microtiter plates to near total confluence in complete culture medium. Multiple uniform and constant streaks were made on the monolayer culture with 10-μl pipette tips. The plates were immediately washed with PBS to remove detached cells. HUVEC cells were incubated with CM obtained from OvCar-3 and PC-3 cells transfected with OPNc or empty vector. Cell migration was monitored for 6 h and photographs were taken at the 0- and 6-h time points. Wound area was calculated for each experimental condition and the percentage of decrease in the wound area, reflecting migration activation, has been calculated using the ImageJ 1.48 software. Triplicate assays has been used to calculate the average percentage of wound area.

For proliferation assays, HUVEC cells (2 × 10^4^, 24-well plates) were cultured with OPNc-CM or EV-CM and proliferation was followed for 24 and 48 h. Cell proliferation was analyzed by crystal violet incorporation assays. For the crystal violet assays, cells were washed twice with PBS and fixed in glutaraldehyde for 10 min, followed by staining with 0.1% crystal violet and solubilization with 0.2% Triton X-100. Microtiter plates were read on a SPECTRAmax GEMINI-XS, using SoftMax Pro software Version 3.1.1.

### Statistical analyses

In all experiments, unless otherwise indicated, the statistical significance of the data was analyzed with a two-tailed, nonpaired or paired Student’s *t* test, using Microsoft Excel Windows software. One way ANOVA test has been used to analyse wound are data. Data are plotted as mean ± SEM. An asterisk (*) denotes *p* < 0.05 and (**) *p* < 0.0001.

## Results

### OPNc modulates the expression of key cancer-related genes in OvCar-3 and PC-3 cells overexpressing this isoform

In order to ascertain the cancer gene pathways modulated by OPNc overexpression in the OvCar-3 and PC-3 cell lines, we performed a qRT-PCR Array analysis of total RNA obtained from OPNc-overexpressing cells, compared to cells transfected with the EV control clone. Using this *in vitro* cell line model, we assessed how OPNc overexpression could modulate different hallmarks of cancer by using the Cancer Pathway Finder Array. This array consisted of 84 genes representing the major biological pathways involved in tumor progression, as described in Methods.

**Figure 1:**
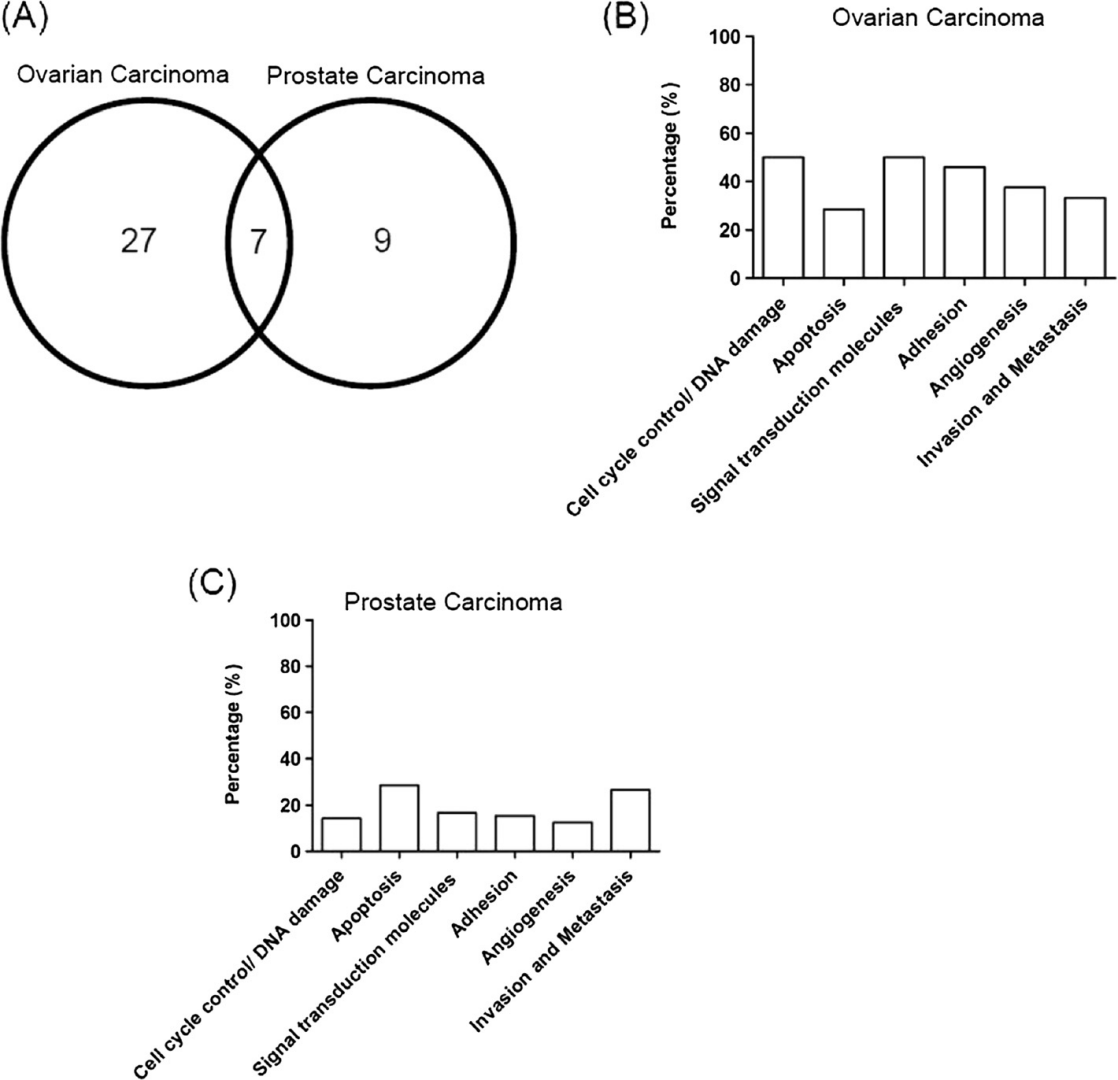
**Venn diagram of overlapping genes with differential expression. (A)** Genes differentially expressed in response to OPNc overexpression among the OC and PCa databases are shown. Cancer functional distribution of the identified genes altered by OPNc overexpression on OC **(B)** and PCa **(C)**. The percentage of differentially expressed genes in each functional class is shown.

A complete list of genes whose expression is significantly modulated by OPNc overexpression in OvCar-3 and PC-3 cells is shown in Additional files 2 and 3. Known roles and clinical implications of these different transcript products in prostate and ovarian tumors are also listed in these files. Among the 84 cancer pathway-focused genes tested, 34 genes in OvCar-3 and 16 genes in PC-3 OPNc-overexpressing cells were differentially expressed when compared to controls (p <0.05 with at least 1.5-fold up- or down-regulation). Among these differentially expressed genes, 27 and 9 of them, respectively, are specifically differentially expressed in OvCar-3 and PC-3 OPNc-overexpressing cells in relation to controls. Venn diagram analysis identified a subset of 7 genes that were differentially expressed in both tumor models (Figure 1A) in relation to control cells. The differentially expressed genes in response to OPNc overexpression in OvCar-3 and PC-3 cells are included in all the major biological pathways involved in tumor progression investigated here. In OvCar-3 cells, a higher percentage of differentially expressed genes are related to cell cycle control and DNA damage repair, apoptosis, and signal transduction molecules and transcription factors (Figure 1B); while in PC-3 cells, a higher percentage is observed for genes related to apoptosis and invasion/metastasis (Figure 1C).

In OvCar-3 OPNc-overexpressing cells, the transcript levels of genes coding for proteins involved in cell cycle control, including *Rb1*, *Cdk2*, *Cdkn1a*, *Ccne1*, *S100a4* and *Cdc25a,* were significantly up-regulated (p <0.05; Additional file 2). Correspondingly, overexpression of OPNc induced the transcript levels of some anti-apoptotic factors, such as *Bcl2l1*, *Bad* and *Casp8*. In this cell line model, the most significant up-regulated genes were those related to invasion and metastasis (*Mmp2* and *Serpine1*), cell adhesion (*Itgb3*) and angiogenesis (*Vegfa*). Only 4 genes were down-regulated in OvCar-3 OPNc-overexpressing cells when compared to EV transfected cells. These genes are related to DNA damage repair (*Atm*), act as transcription factors (*Fos* and *Myc*), or perform important roles in cancer-cell invasion (*Mmp1*).

PC-3 OPNc-overexpressing cells showed a different transcriptional pattern, compared to OvCar-3 overexpressing this isoform (Additional file 3). The most significant up-regulated genes identified in this tumor model are related to chromosome-end replication (*Tert*), invasion and metastasis (*Plau*, *Serpine1*, *Mmp9* and *Mmp1*), cell adhesion (*Itgb3* and *Itgav*) and angiogenesis (*Angpt1* and *Vegfa*). Down-regulation in PC-3 cells as a result of OPNc overexpression was observed for *Htatip2* (a gene related to apoptosis control) and for *Fos*, a transcription factor-coding gene. The specific transcriptional patterns observed for both OvCar-3 and PC-3 OPNc-overexpressing cells revealed the potential of this splice variant to contribute to all the main acquired capabilities required for tumor progression, although each of these tumor-cell types evokes different signaling pathways.

Genes that are commonly modulated as a result of OPNc overexpression in both OvCar-3 and PC-3 cells (*Bcl2l1*, *Bad*, *Fos*, *Itgav*, *Itgb3*, *Vegfa* and *Serpine1*) (Additional files 2 and 3) are presumably required for shared functions related to cancer progression in both tumor models. Taken together, these data indicate that OvCar-3 and PC-3 OPNc-overexpressing cells modulate specific transcriptional patterns related to key aspects of cancer progression, although part of these signaling pathways is commonly regulated in both cell-line tumor models.

### Functional interaction networks for the OPNc-signature genes

**Figure 2:**
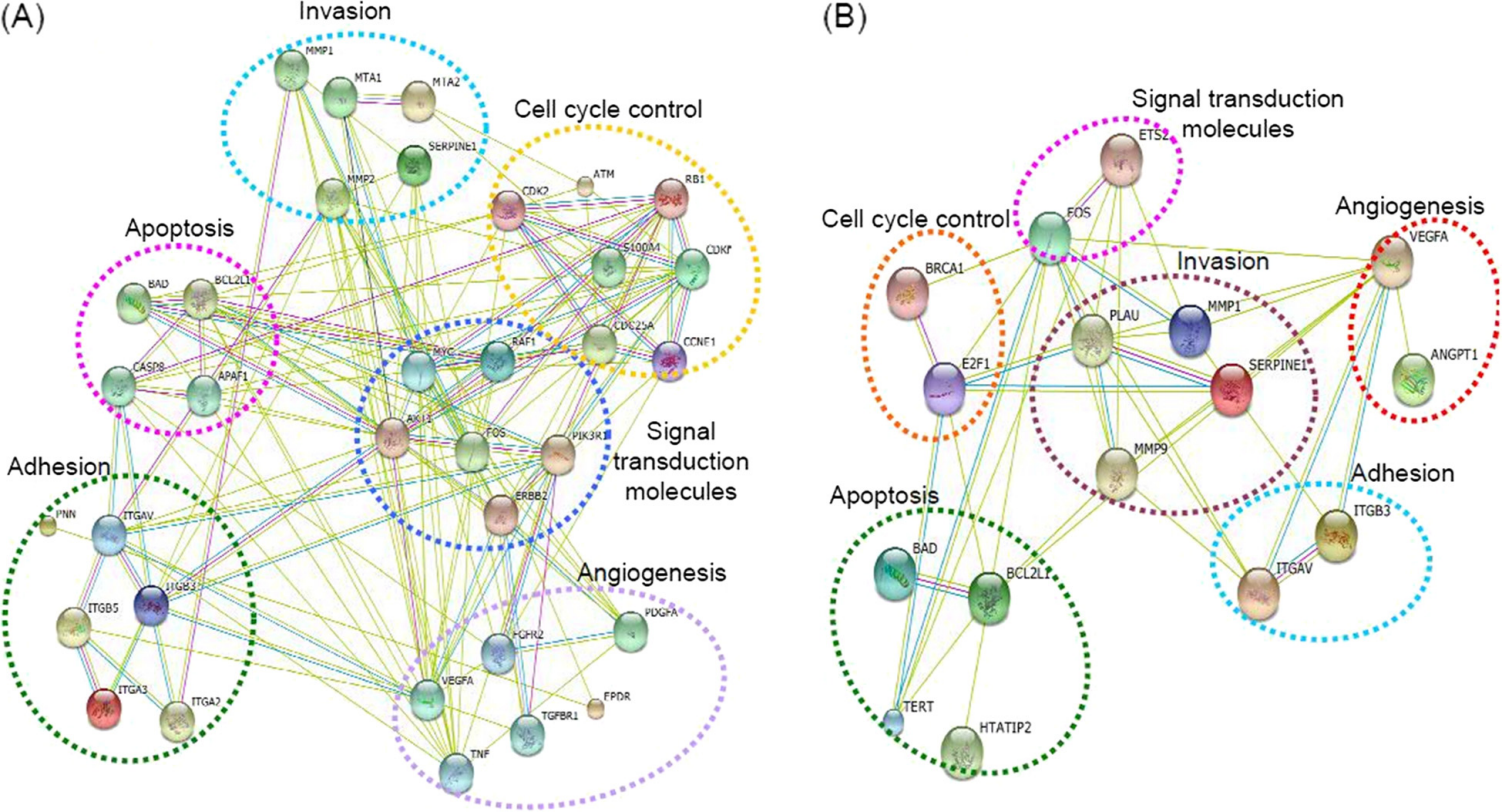
**The cancer functional interaction network among the genes induced by OPNc overexpression in ovarian (A) and prostate carcinoma model (B).** To model an interaction network, STRING 9.0 software was used, where a node represents a protein and a line represents a protein-protein interaction. The network modules were manually dissected based on the network structure and gene functions, with their most concordant functions labeled above them. **(A)** The connected component of the OC-OPNc induced gene network, representing 34 proteins and 132 interactions among them. **(B)** In PCa, there are 16 altered proteins with 36 interactions among them. Green, red, blue, black, pink, light blue, brown and purple lines indicate neighborhood, gene fusion, co-occurrence, coexpression, experiments, databases, text-mining and homology, respectively.

A gene interaction network was constructed by using the differentially expressed genes in OvCar-3 and PC-3 OPNc-overexpressing cells and controls, as described in Methods. A dataset containing the differentially expressed genes, called the focus molecules, between ovarian and prostate-cancer cell lines and controls was overlaid onto a global molecular network developed from information contained in the STRING database, which contains known and predicted protein interactions. The interactions include direct (physical) and indirect (functional) associations, and are derived from four sources: genomic context, high-throughput experiments, (conserved) co-expression and previous knowledge. The network contains statistically significant deregulated genes and putative interacting proteins. In particular, the signature genes that were differentially expressed in OvCar-3 and PC-3 OPNc-overexpressing cells were largely enclosed by six network modules (Figure 2A and 2B). The resulting networks were observed to be highly modular and tightly connected modules, indicating OPNc as a driver event activating cancer-hallmark-related pathways. In the OvCar-3 OPNc-overexpressing cell network, we identified 132 putative interactions among 34 proteins coded by transcripts that are up- or down-regulated in response to OPNc-overexpression, indicating that multiple signaling pathways related to cell proliferation, the cell cycle and signal transduction molecules might be constitutively activated through genomic amplification of these key regulators. This in turn indicates promotion of tumor cell proliferation, thus contributing to the poor prognosis of OC (Figure 2A). To determine whether the 16 OPNc-regulated proteins in PC-3 cells were functionally related, we also generated a network map of interactions. We then found 36 potential interactions, among 16 proteins coded by transcripts modulated by OPNc-overexpression (Figure 2B). The enriched interconnected PPIs within these modules might imply massive crosstalk among regulators of apoptosis, invasion and metastasis, suggesting that multiple signaling pathways related to these hallmarks might be constitutively activated through these regulators. These data further suggest that OPNc exerts its effects on tumor progression features through networks associated with cancer hallmarks.

### Conditioned medium secreted by OPNc-overexpressing cells induces endothelial cell adhesion, proliferation and migration

The data obtained here demonstrated that *Vegfa* is one of the most up-regulated transcripts in response to OPNc overexpression in both OvCar-3 and PC-3 cells (Additional files 2 and 3). Also, OvCar-3 and PC-3 OPNc-overexpressing cells and xenograft tumors formed by these cells up-regulate the expression of *Vegf* transcript [6,8]. We also previously demonstrated that CM secreted by OPNc-overexpressing cells is able to stimulate several aspects of ovarian and prostate cancer progression [6,8] and that CM secreted from OvCar-3 cells overexpresses the VEGF protein in relation to CM secreted from EV transfected cells (data not shown). Considering that some OPNc specifically-responsive transcripts code for secreted proteins and that some of these gene products include those mediating OPNc pro-tumorigenic properties, we used CM secreted by OPNc-overexpressing cells to partially validate its related transcriptional profiling. Since we observed here that *Vegf* is one of the most up-regulated OPNc-induced genes and VEGFA is one of the earliest and a key mediator of angiogenesis [13], we attempted to validate part of the data obtained in this Cancer Gene Array by testing the effect of OPNc-conditioned medium (OPNc-CM) on different aspects of angiogenesis stimulation.

**Figure 3:**
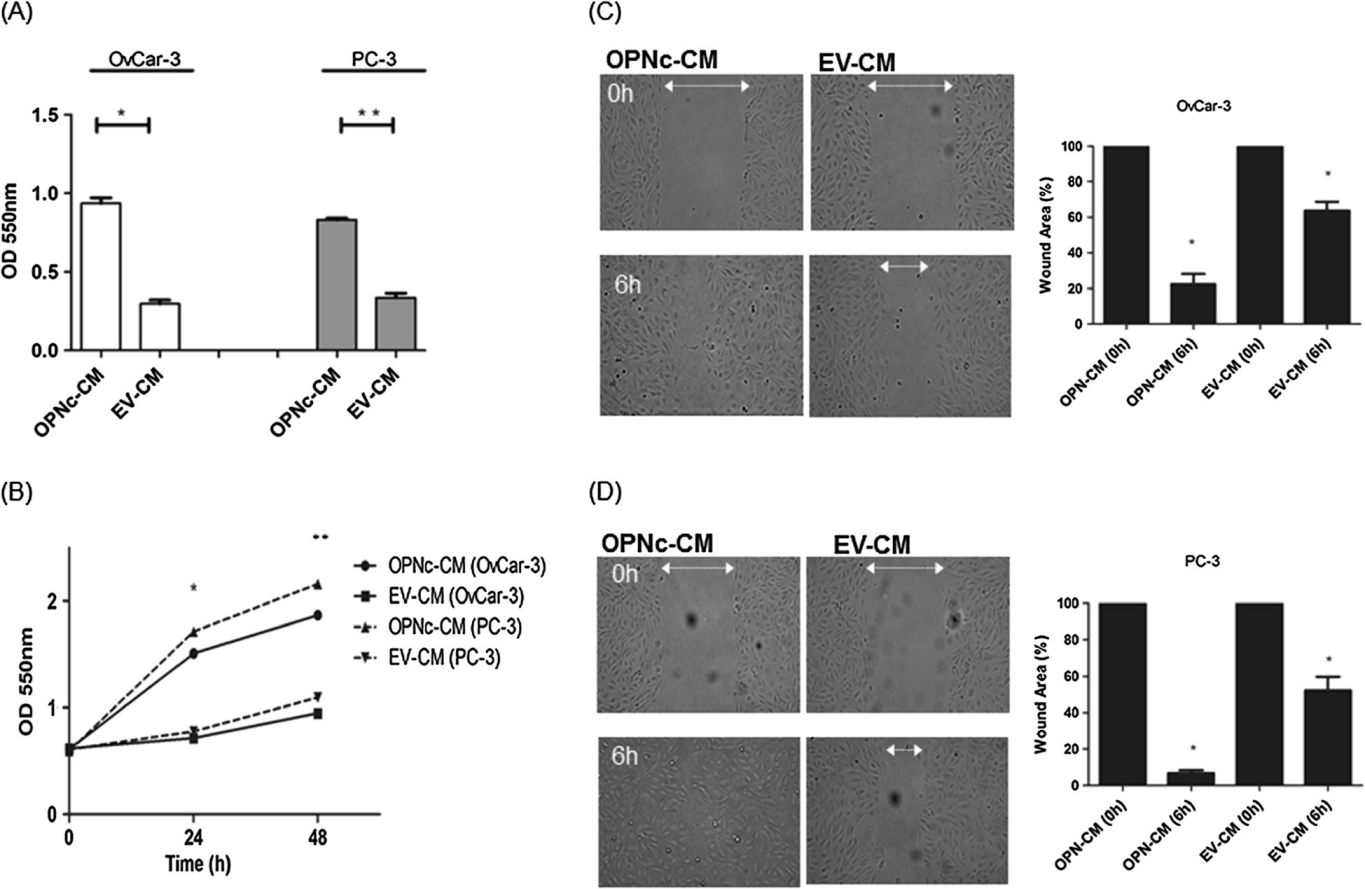
**OPNc-CM from OvCar-3 and PC-3 cells induces adhesion, proliferation and migration of HUVEC cells. (A)** Endothelial cells were plated onto OPNc and EV-conditioned medium (CM) to evaluate cell adhesion. Cells were allowed to adhere for 2 hours and were quantified as described in the Methods section. Error bars represent the mean SD of 3 independent experiments. O.D., optical density measured at 550 nm. **p < 0.0001. **(B)** HUVECs were cultured with OPNc and EV-CM. Proliferation kinetics were evaluated by crystal violet staining. Error bars represent the mean SD of 3 independent experiments. *p < 0.05. O.D., measured at 550 nm. **(C)** and **(D)** OPNc-CM from OvCar-3 and PC-3 cells induces higher HUVEC cell migration rates. HUVEC cells were plated as indicated in the Methods section and analyzed for cell migration by calculating wound area, which was assessed after 6 h. Phase-contrast photomicrographs were taken at 0 and 6 h after migration was initiated. Wound margins are shown at time points 0 (upper panels) and 6 hours (lower panels). The columns show the% of mean wound area ± SD for three independent experiments. *p < 0,05.

Angiogenesis is a multistep process that activates the migration and proliferation of endothelial cells in the perivascular stroma in order to form new capillary vessels. During this process, these sprouting cells stop proliferating and then adhere, align, form tubes, and finally produce new operational vessels [14]. We then investigated whether OPNc-CM, containing overexpressed VEGF, is involved in this multistep angiogenic process, by studying its effect on adhesion, proliferation and migration in human umbilical vein endothelial cells (HUVECs).A higher proportion of HUVEC adhered cells was observed when using OPNc-CM secreted from OvCar-3 and PC-3 cells as a coating substrate, compared to conditioned medium secreted from EV transfected cells (EV-CM) (p <0.0001) (Figure 3A).We next asked whether the growth rates of HUVEC cells cultured with OPNc-CM were altered compared to EV-CM secreted by OvCar-3 and PC-3 cells. As shown in Figure 3B, OPNc-CM secreted from both OvCar-3 and PC-3 cells significantly activated HUVEC proliferation rates, compared to EV-CM in the range of 0 – 48 h of cell culture (p < 0.05).The effect of OPNc-CM on modulating the migration of endothelial cells was also tested, by evaluating the migration of HUVEC cells when cultured in OPNc-CM or EV-CM secreted by OvCar-3 and PC-3 cells. HUVEC cells cultured in OPNc-CM produced by both OvCar-3 and PC-3 cells showed a higher migration rate than HUVEC cells cultured in EV-CM (Figure 3C and 3D). The effect of OPNc-CM in inducing HUVEC cell migration was 50% and 40% higher than that of EV-CM secreted by OvCar-3 and PC-3 cells, respectively.

In summary, these data indicate that OPNc-CM could act as a pro-angiogenic factor for HUVEC cells, and that this CM can significantly affect different key aspects of the early angiogenic process. In addition, these data validate part of the gene-expression patterns induced by OPNc overexpression, which significantly up-regulated *Vegf* in both OvCar-3 and PC-3 cell line tumor models.

## Discussion

Although several reports have demonstrated the important role of full-length OPN in modulating tumor progression [1], only a few studies have identified its gene expression-related profiling [5,9]. This is the first report regarding gene expression data related to a specific OPN splice variant and correlation with its functional roles in tumor progression. Although other reports have pointed to individual pathways modulating the actions of some OPN splice variants in different tumor models [5], none of them has investigated putative interacting signaling networks and their relationships to functional data. The main finding of the current study is that OPNc splice variant overexpression can modulate key cancer pathways and gene transcriptional patterns associated with the progression of ovarian and prostate tumors. In total, changes in the expression of 34 genes in OvCar-3 and of 16 genes in PC-3 OPNc-overexpression cells were observed, with an overlap of 7 genes (8.3%) in the two cell lines (Figure 1A). Gene ontology and interactome analysis led to a categorization of our data set into functional categories and networks (Additional files 2 and 3, and Figure 2). The array data revealed that OPNc overexpression modulates the expression of genes related to cell cycle control, apoptosis, signal transduction molecules and transcription factors, adhesion, angiogenesis, invasion and metastasis in both cell-line tumor models. These data were correlated with previously described roles of OPNc in activating tumor progression. Among the significantly up-regulated genes identified were *Vegf* and other pro-angiogenic gene products. Based on these data, we could partially validate the results obtained here, using gene expression profiling to demonstrate that OPNc-overexpressing cells secrete factors that are able to activate early angiogenic processes.

### Transcriptional patterns associated with cell cycle control, proliferation and apoptosis

Previous studies from our group demonstrated that OPNc favors the proliferation of ovarian and prostate carcinoma cells [6,8]. Here, we identified several differentially expressed cell proliferation-related genes in both tumor cell line models as a result of OPNc overexpression. In OvCar-3 OPNc-overexpressing cells, *Atm* was significantly down-regulated, and 6 other genes were up-regulated (*Rb1*, *Cdk2*, *Cdkn1a*, *Ccne1*, *S100a4* and *Cdc25a).* The deregulated patterns of these genes may be related to the progression of ovarian and prostate cancer, according to the known roles for these genes, as presented in Additional files 2 and 3 [15-25].

We also previously showed that OvCar-3 and PC-3 OPNc-overexpressing cells that were treated with the anti-OPNc antibody proliferated more slowly and were induced to die, further evidencing a survival role for OPNc [6,8]. In this study we found that *Bcl2l1* and *Bad* were up-regulated in OvCar-3 and PC-3 OPNc-overexpressing cells compared to control cells. Consistently with our findings, these gene products have been reported as involved in the survival and chemosensitivity of prostate and ovarian carcinoma cells [15-30]. We also observed a significant upregulation of *Casp8* and *Apaf1,* which have been implicated in death-receptor-mediated apoptosis and chemoresistance [24,25]. In PC-3 cells, we also identified *Htatip2* and *Tert* deregulated expression in response to OPNc overexpression, which are also gene products that mediate cell survival, metastasis and cancer recurrence [28,30].

### Transcriptional patterns associated with signal transduction and transcription factors

The results of this study are also in accordance with previous data demonstrating pathways related to signal transduction and transcription factors that are typically activated in ovarian and prostate tumor progression [31,32]. We have shown that PI3K/Akt has an important pro-survival role and mediates several pro-tumorigenic features evoked by OPNc overexpression in OvCar-3 and PC-3 cells [6,8]. Our current data provide evidence of the existence of additional deregulated signal transduction pathways and transcription factors in OvCar-3 and PC-3 cells as a result of OPNc overexpression. The *Fos* gene was found to be down-regulated in response to OPNc overexpression in OvCar-3 and PC-3 cells. Specifically in OvCar-3 OPNc-overexpressing cells, down-regulation of *Myc* and up-regulation of *Pik3r1*, *Raf1*, *Erbb2* and *Akt1* genes were observed. It has been reported that the tumor environment down-regulates c-MYC protein levels, which might be a strategy for cancer cells to survive under conditions of limited energy resources [33]. However, down-regulation of c-MYC has not been described previously in ovarian carcinoma cells. Additional clinical implications of differentially expressed genes able to modulate signaling pathways and transcription are listed in Additional files 2 and 3 [34-40].

### Transcriptional patterns associated with cell adhesion and angiogenesis

Regarding gene expression patterns related to cancer-associated adhesion molecules, in OvCar-3 cells, in addition to up-regulation of integrins in response to OPNc overexpression, we found an up-regulation of Pinin (*Pnn*). The up-regulation of a number of integrin heterodimers and adhesion molecules in cells that constitutively overexpress OPNc may therefore represent additional mechanisms by which cells acquire a general ability to adhere, promoting ovarian and prostate tumor progression, consistent with the well-known integrin-mediated role of total OPN, especially in cancer cells [41].

Previously, we have also shown that OPNc significantly increases *Vegfa* mRNA in ovarian carcinoma and prostate cancer xenograft tumors [6,8]. Here, we also observed *Vegfa* up-regulation in response to OPNc-overexpression in OvCar-3 and PC-3 cells. Published reports have also indicated that VEGF-A is overexpressed in ovarian carcinoma and prostate cancer, and has been associated with tumor growth and recurrence [42,43]. In addition to *Vegfa* overexpression, we found that OvCar-3 OPNc-overexpressing cells up-regulate *Epdr1*, *Pdgfa*, *Tgfbr1*, *Tnf* and *Fgfr2*, all of which are able to directly or indirectly modulate different aspects of angiogenesis, such as vascular permeability, lymphatic metastasis and tumor-stroma interactions, endothelial cell survival and stable vasculature [42-52].

Based on the significant up-regulation of several pro-angiogenic transcripts in response to OPNc overexpression, we attempted to validate part of the data obtained here by investigating the effect of OPNc-CM on activating angiogenic properties. Our data clearly demonstrated that OPNc-CM activated different steps of early angiogenesis, such as endothelial cell proliferation, adhesion and migration. In the light of data previously published by our group and those presented here, we partially validated that OvCar-3 and PC-3 OPNc-overexpressing cells secrete specific proteins that create a permissive environment, favoring induction of their own angiogenesis. However, the specific factors or proteins mediating these pro-angiogenic features contained in this CM should be further validated and characterized. OPN has been broadly characterized as an inducer of tumor angiogenesis, with a particular correlation with VEGF expression [53,54]. The specific actions of OPN splice variants regarding non-small-cell lung cancer angiogenesis and VEGF have been investigated [55]. These authors showed that OPNa overexpression was associated with increased bovine capillary endothelial tubule length and VEGF secretion, whereas OPNc was associated with decreases in both. OPNc in this tumor model has opposite roles to the OPNc-induced angiogenic features that we observed in ovarian and prostate carcinoma cells. Considering the tumor-specific roles of the OPN splice variants, the means by which different splicing isoforms specifically modulate tumor angiogenesis should be further investigated.

Previously, we have also shown that OPNc overexpression stimulates OvCar-3 and PC-3 migration, invasion and the formation of colonies in semisolid medium. OvCar-3 and PC-3 cells overexpressing OPNc resulted in extremely rapid tumor growth *in vivo*. In these tumors, well-known markers of tumor progression able to modulate tumor invasion and metastatic potential, such as *Mmp2* and *Mmp9,* were consistently up-regulated [6,8]. Transcriptional levels of pro-metastatic genes such as *Mmp1* and *Serpine1* in both OvCar-3 and PC-3 cells; *Mta1, Mta2* and *Mmp2* in OvCar-3; and *Plau* and *Mmp9* in PC-3 cells, were significantly modulated in OPNc-overexpressing cells. However, our present study provides the first indication that OPNc might down-regulate *Mmp1* in OC. The literature has shown the involvement of these transcripts in the steps modulating tumor invasion and metastasis in both ovarian and prostate tumors [56-63]. Here, also consistent with our previous data [6,8], we report that OPNc-overexpressing cells induce *Mmp2* and *Mmp9* overexpression in ovarian carcinoma and prostate cancer cells, respectively, further corroborating a role of these gene products in activating cell invasion in both tumor models [60,62].

#### ***Interacting networks and associated transcriptional patterns***

The existence of an interactome network indicates that these differentially expressed genes, besides inducing specific cancer-associated pathways, potentially interact with each other, further indicating that the roles of OPNc in activating ovarian and prostate cancer progression require multiple and crosstalk signaling. The different network patterns observed for each tumor model investigated here concord with previously discussed tissue- and tumor-specific roles for OPNc and other OPN splice variants [5]. Further studies aimed at exploring the mechanisms by which OPNc modulates these target and interacting gene products will elucidate how it controls the proliferation of ovarian and prostate cancer cells.

## Conclusions

In conclusion, we have shown here that OvCar-3 and PC-3 cells that constitutively overexpress OPNc induce an altered gene expression profile that reflects the main acquired capabilities of cancer. We have also shown, for the first time, that conditioned medium secreted by these OPNc-overexpressing cells contains factors that are able to induce early angiogenic processes, partially validating the array data. Taken together, these data not only support our previously characterized OPNc pro-tumorigenic cellular functions, but also suggest that given the diversity of genes for which OPNc is able to regulate expression, it is possible that OPNc signaling may be a key regulatory circuit that dictates cell physiology during the progression of these tumors. Further work is required to functionally validate additional cancer molecular mechanisms stimulated by OPNc in both tumor models. Finally, this study provides a framework for the identification of key contributors to malignancy, and may lead to new insights useful in the development of therapeutic interventions for ovarian and prostate cancer treatment and prevention.

## Abbreviations

OPNc: Osteopontin-c; CM: Conditioned medium; OPN-SI: OPN Splicing isoforms; EV: Empty vector; PPI: Protein–protein interaction; OPNc-CM: CM Produced by OPNc-overexpressing cells; EV-CM: CM Produced by EV controls; qRT-PCR: Quantitative real-time PCR.

## Competing interests

The authors declare that they have no competing interests.

## Authors’ contributions

TM, AB and EG analyzed and interpreted the data. TM collected data. TM and EG drafted the manuscript. TM, AB, VC and EG contributed to the conception and design of the study. All authors read and approved the final manuscript.

## Pre-publication history

The pre-publication history for this paper can be accessed here:

http://www.biomedcentral.com/1471-2407/14/433/prepub

## Supplementary Material

Additional file 1 **Splicing isoforms of OPN (OPN-SI) are overexpressed in OvCar-3 (A and B) and PC-3 (C and D) cell lines.** (A and C) OPN-SI transcript levels were analyzed by qRT-PCR and were represented by relative expression level in relation to empty vector-transfected cells. GAPDH was used as a normalization control. (B and D) Immunoblot analysis of total intracellular and secreted protein extracts from OPN-SI overexpression clones using the incubated O/N with the human anti-OPNc primary antibody, demonstrating the overexpression of OPNc (around 55 KDa), OPNb (around 60 KDa), and OPNa (around 72 KDa) protein isoforms in each corresponding overexpression clone, respectively. OvCar-3 and PC-3 cells overexpressing clones have been compared to cell extracts transfected with an empty vector control clone. OPN-SI molecular weights vary, according to post-translational modifications, which are cell type-dependent. Click here for file

Additional file 2 **Genes differentially expressed in OvCar-3 cells overexpressing OPNc.** Multiple genes related to cell cycle control and DNA damage repair, apoptosis, signal transduction and gene regulation, cell adhesion, angiogenesis, invasion and metastasis were evaluated for expression levels using the RT2 Profiler PCR Array system. This table lists genes that showed significant delta CT (p < 0.05) values, and genes with at least a 1.5-fold change in gene expression levels in OPNc-overexpressing cells relative to empty vector (EV) OvCar-3 transfected cells. Positive values indicate up-regulation of individual genes; negative values indicate down-regulation. Roles of each gene were drawn from literature references on ovarian carcinoma. The data were evaluated by two-tailed Student’s t test. *OPNc - commonly modulated genes in both OvCar-3 and PC-3 carcinoma models [15-25,33-38,42,44-50,56-60]. Click here for file

Additional file 3 **Genes differentially expressed in PC-3 cells overexpressing OPNc.** Multiple genes related to cell cycle control and DNA damage repair, apoptosis, signal transduction and gene regulation, cell adhesion, angiogenesis, invasion and metastasis were evaluated for expression levels using the RT2 Profiler PCR Array system. This table lists genes that show significant delta CT (p < 0.05) values and genes with at least a 1.5-fold change in gene expression levels in OPNc-overexpressing cells, relative to empty vector (EV) PC-3 transfected cells. Roles of each gene were drawn from literature references on prostate carcinoma. Positive values indicate up-regulation of individual genes; negative values indicate down-regulation. The data were evaluated by two-tailed Student’s t test. *OPNc - commonly modulated genes in both OvCar-3 and PC-3 carcinoma models [22,26-30,39,40,43,51,52,59,61-63]. Click here for file
