# Supplementary figures and images for: Changes in the transcriptional profile in response to overexpression of the osteopontin-c splice isoform in ovarian (OvCar-3) and prostate (PC-3) cancer cell lines
Source: BMC Cancer. 2014 Jun 13;14:433. doi: 10.1186/1471-2407-14-433 (PMC4075779; doi:10.1186/1471-2407-14-433)

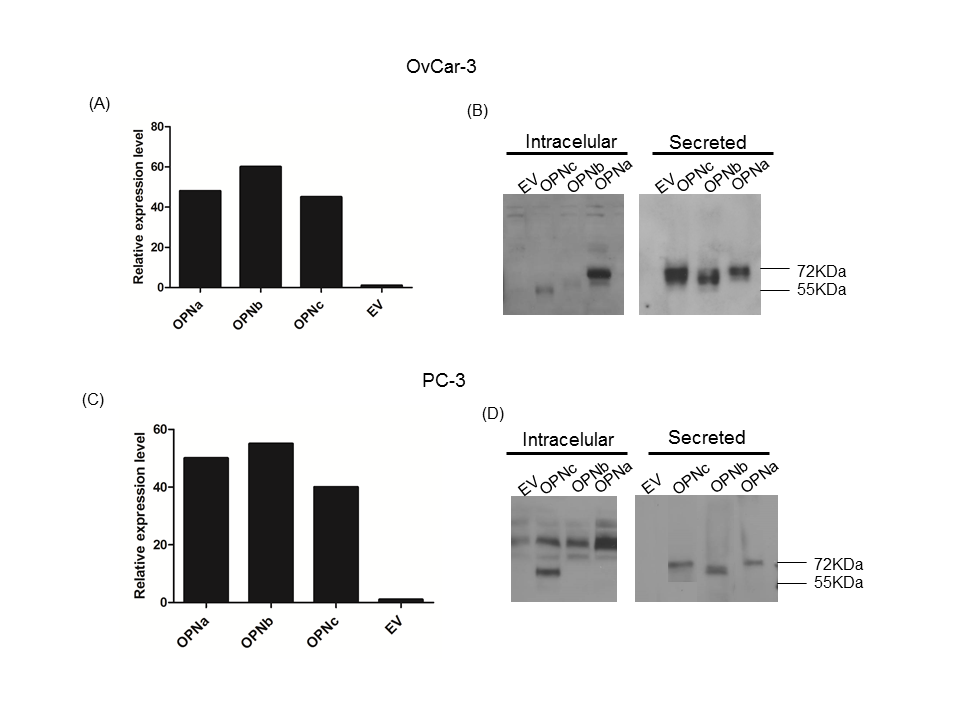

Supplement: Additional file 1 — Splicing isoforms of OPN (OPN-SI) are overexpressed in OvCar-3 (A and B) and PC-3 (C and D) cell lines. (A and C) OPN-SI transcript levels were analyzed by qRT-PCR and were represented by relative expression level in relation to empty vector-transfected cells. GAPDH was used as a normalization control. (B and D) Immunoblot analysis of total intracellular and secreted protein extracts from OPN-SI overexpression clones using the incubated O/N with the human anti-OPNc primary antibody, demonstrating the overexpression of OPNc (around 55 KDa), OPNb (around 60 KDa), and OPNa (around 72 KDa) protein isoforms in each corresponding overexpression clone, respectively. OvCar-3 and PC-3 cells overexpressing clones have been compared to cell extracts transfected with an empty vector control clone. OPN-SI molecular weights vary, according to post-translational modifications, which are cell type-dependent. [file 1471-2407-14-433-S1.tiff]
