# Supplementary material for: Changes in the transcriptional profile in response to overexpression of the osteopontin-c splice isoform in ovarian (OvCar-3) and prostate (PC-3) cancer cell lines
Source: BMC Cancer. 2014 Jun 13;14:433. doi: 10.1186/1471-2407-14-433 (PMC4075779; doi:10.1186/1471-2407-14-433)
Supplement: Additional file 2 — Genes differentially expressed in OvCar-3 cells overexpressing OPNc. Multiple genes related to cell cycle control and DNA damage repair, apoptosis, signal transduction and gene regulation, cell adhesion, angiogenesis, invasion and metastasis were evaluated for expression levels using the RT2 Profiler PCR Array system. This table lists genes that showed significant delta CT (p < 0.05) values, and genes with at least a 1.5-fold change in gene expression levels in OPNc-overexpressing cells relative to empty vector (EV) OvCar-3 transfected cells. Positive values indicate up-regulation of individual genes; negative values indicate down-regulation. Roles of each gene were drawn from literature references on ovarian carcinoma. The data were evaluated by two-tailed Student’s t test. *OPNc - commonly modulated genes in both OvCar-3 and PC-3 carcinoma models [15-25,33-38,42,44-50,56-60]. [file 1471-2407-14-433-S2.doc]

**Additional file 2. Genes differentiated expressed in OvCar-3 cells overexpressing OPNc.**

|  |  |  |  | **Ovarian Carcinoma** | |
| --- | --- | --- | --- | --- | --- |
| **Function** | **Gene** | **Refseq** | **Notes** | **Fold-change**  **(OPNc X EV)** | **p value** |
| **Cell cycle control and DNA damage repair** |  |  |  |  |  |
|  | *Atm* | NM_000051 | *Atm* mutations confer increased susceptibility to OC [15]. | -4.72 | 0.001434 |
|  | *Rb1* | NM_000321 | *Rb1, a* classical tumor suppressor gene, methylates SMYD2, stimulating cell cycle progression [16]. | +1.8 | 0.024487 |
|  | *Cdk2* | NM_001798 | *Cdk2* overexpression is observed in ovarian cancer samples [17]. | +1.85 | 0.034487 |
|  | *Cdkn1a* | NM_000389 | *Cdkn1a* overexpression promotes OC cell migration, invasion and cell proliferation [18]. | +2.07 | 0.019299 |
|  | *Ccne1* | NM_001238 | Cyclin E overexpression is related to mechanisms of stimulation of cell proliferation in ovarian carcinoma [19]. | +2.3 | 0.003111 |
|  | *S100a4* | NM_002961 | S100A4 function in cell cycle progression and has been associated with metastasis formation and poor prognosis in ovarian carcinoma [20]. | +2.66 | 0.011249 |
|  | *Cdc25a* | NM_001789 | High CDC25A expression is related to a worse prognosis in OC patients [21]. | +2.8 | 0.000906 |
| **Apoptosis** |  |  |  |  |  |
|  | *Bcl2l1** | NM_138578 | Bcl2-like 1 inhibits apoptosis by blocking the translocation of BAX to the mitochondrial outer membrane [22]. | +2.17 | 0.020561 |
|  | *Bad** | NM_004322 | *Bad* is a death-promoting BH3-only member of the BCL-2 family. Paclitaxel-resistant ovarian carcinoma cells contain high levels of phospho-BAD [23]. | +2.44 | 0.003939 |
|  | *Casp8* | NM_001228 | In cisplatin-resistant OC cells, an induction of caspase 8 protein expression is the key factor of TRAIL sensitization [24]. | +2.86 | 0.022621 |
|  | *Apaf1* | NM_001160 | In OC cells, *Apaf1* gene seems to be activated, which may contribute both to the pathogenesis of OC and to chemoresistance [25]. | +3.84 | 0.013762 |

| **Signal transduction molecules and transcription factors** |  |  |  |  |  |
| --- | --- | --- | --- | --- | --- |
|  | *Fos** | NM_005252 | Loss of c-Fos expression is associated with tumour progression in OC and may be a prognostic factor [34] | -2.12 | 0.001039 |
|  | *Myc* | NM_002467 | c-MYC down-regulation might be a strategy for cancer cells to survive under conditions of limited energy sources [33]. | -1.82 | 0.008713 |
|  | *Pik3r1* | NM_181504 | PI3K p85 subunit is overexpressed and positively correlated to OC progression [35]. | +1.65 | 0.003498 |
|  | *Raf1* | NM_002880 | *Raf1* has been associated with OC cell survival, by stimulating cell proliferation and inhibiting apoptosis [36]. | +1.73 | 0.008429 |
|  | *Erbb2* | NM_004448 | The *Erbb2* (*Her2/Neu*) receptor is overexpressed in 9-30% of OC and is correlated with poor prognosis and chemoresistance [37]. | +2.46 | 0.00272 |
|  | *Akt1* | NM_005163 | Overactivation of AKT1 is frequently detected in OC [38]. | +2.56 | 0.002432 |
| **Adhesion** |  |  |  |  |  |
|  | *Pnn* | NM_002204 | *Pnn* is overexpressed in OC specimens and can modify the co-repressor function of C-terminal binding protein 2 (CtBP2), which is an OC oncogene [44]. | +1.85 | 0.028181 |
|  | *Itga3* | NM_002687 | The classic literature regarding cancer and integrins has implicated this family as adhesion receptors related to cell proliferation, migration and survival [45]. | +1.74 | 0.046677 |
|  | *Itgav** | NM_002210 |  | +1.91 | 0.002056 |
|  | *Itgb5* | NM_002213 |  | +2.85 | 0.007166 |
|  | *Itga2* | NM_002203 |  | +3.44 | 0.000838 |
|  | *Itgb3** | NM_000212 |  | +5.15 | 0.047081 |
| **Angiogenesis** |  |  |  |  |  |
|  | *Epdr1* | NM_017549 | Ependymin related protein 1 may play a role in calcium-dependent cell adhesion [46]. | +1.5 | 0.008205 |
|  | *Pdgfa* | NM_002607 | *Pdgf* induces cell growth, survival, transformation, migration and vascular permeability and has also been described as a useful marker for OC [47]. | +1.54 | 0.022463 |
|  | *Tgfbr1* | NM_004612 | Activated TGFBR1 mediates invasion and metastasis of OC cells [48]. | +1.57 | 0.009081 |
|  | *Tnf* | NM_000594 | TNF-α induce myeloid cell recruitment into the tumor microenvironment and enhance OC growth [49]. | +1.83 | 0.032322 |
|  | *Fgfr2* | NM_000141 | Gene amplification or missense mutation of *Fgfr2* occurs in OC [50]. | +2.34 | 0.001965 |
|  | *Vegfa** | NM_003376 | VEGFA is overexpressed in OC and has been associated with increased tumor growth, ascites fluid accumulation, metastasis, poor prognosis and reduced survival [42]. | +51.53 | 0.005972 |
| **Invasion and metastasis** |  |  |  |  |  |
|  | *Mmp1* | NM_002421 | MMP-1 is often associated with poor survival in OC, by stimulating degradation of extracellular matrix, enhancing the progression of ovarian cancers [56]. | -2.18 | 0.045249 |
|  | *Mta1* | NM_004689 | *Mta1 has* an important role in the OC progression, by enhancing anchorage-independent cell growth in semisolid medium [57]. | +1.56 | 0.021296 |
|  | *Mta2* | NM_004739 | *Mta2* overexpression is associated with aggressive behavior of epithelial OC [58]. | +2.33 | 0.038178 |
|  | *Serpine1** | NM_000602 | *Serpine1* overexpression is associated with a poor prognosis and reduced disease-free survival in patients with OC, by maintaining an angiogenic ‘scaffold’ and stabilizing nascent capillary structure [59]. | +6.44 | 0.010252 |
|  | *Mmp2* | NM_004530 | *Mmp2* is overexpressed in advanced OC [60]. | +611.98 | 0.001808 |

Multiple genes related to Cell cycle control and DNA damage repair, apoptosis, signal transduction and gene regulation, cell adhesion, angiogenesis, invasion and metastasis were evaluated for expression levels using the RT2 Profiler PCR Array system. This table lists genes that show significant delta CT (p< 0.05) values, and genes with at least 1.5-fold change in gene expression levels in OPNc-overexpressing cells relative to empty vector (EV) OvCar-3 transfected cells. NM indicates gene bank accession numbers. Positive values indicate up-regulation of individual genes; negative values indicate down-regulation. Roles of each gene were drawn from ovarian carcinoma literature. The data were evaluated by two-tailed Student’s t test. *OPNc-commonly modulated genes in both OvCar-3 and PC-3 carcinoma models.
