# Supplementary material for: Changes in the transcriptional profile in response to overexpression of the osteopontin-c splice isoform in ovarian (OvCar-3) and prostate (PC-3) cancer cell lines
Source: BMC Cancer. 2014 Jun 13;14:433. doi: 10.1186/1471-2407-14-433 (PMC4075779; doi:10.1186/1471-2407-14-433)
Supplement: Additional file 3 — Genes differentially expressed in PC-3 cells overexpressing OPNc. Multiple genes related to cell cycle control and DNA damage repair, apoptosis, signal transduction and gene regulation, cell adhesion, angiogenesis, invasion and metastasis were evaluated for expression levels using the RT2 Profiler PCR Array system. This table lists genes that show significant delta CT (p < 0.05) values and genes with at least a 1.5-fold change in gene expression levels in OPNc-overexpressing cells, relative to empty vector (EV) PC-3 transfected cells. Roles of each gene were drawn from literature references on prostate carcinoma. Positive values indicate up-regulation of individual genes; negative values indicate down-regulation. The data were evaluated by two-tailed Student’s t test. *OPNc - commonly modulated genes in both OvCar-3 and PC-3 carcinoma models [22,26-30,39,40,43,51,52,59,61-63]. [file 1471-2407-14-433-S3.doc]

**Additional file 3. Genes differentiated expressed in PC-3 cell overexpressing OPNc.**

|  |  |  |  | **Prostate Carcinoma** | |
| --- | --- | --- | --- | --- | --- |
| **Function** | **Gene** | **Refseq** | **Notes** | **Fold-change**  **(OPNc X EV)** | **p value** |
| **Cell cycle control and DNA damage repair** |  |  |  |  |  |
|  | *E2f1* | NM_005225 | *E2f1* is upregulated in metastatic tissues from hormone-resistant PCa patients, and is involved in resistance to drug-induced apoptosis [26]. | +2.04 | 0.034428 |
|  | *Brca1* | NM_007294 | Overexpression of *Brca1* may provide a survival signal for PCa growth in the presence of normal *Brca1* [27]. | +2.38 | 0.042483 |
| **Apoptosis** |  |  |  |  |  |
|  | *Htatip2* | NM_006410 | *Htatip2* (TIP30) nuclear expression has been associated with PCa progression and metastasis [28]. | -5.77 | 0.00973 |
|  | *Bad** | NM_004322 | BAD provides a proliferative advantage to prostate tumors [29]. | +2.03 | 0.030344 |
|  | *Bcl2l1** | NM_138578 | Bcl2-like 1 inhibits apoptosis by blocking the translocation of BAX to the mitochondrial outer membrane [22]. | +2.5 | 0.043878 |
|  | *Tert* | NM_198253 | *Tert* can be a valuable tool for early prediction of PCa biochemical recurrence after radical prostatectomy [30]. | +15.65 | 0.046841 |
|  |  |  |  |  |  |
| **Signal transduction molecules and transcription factors** |  |  |  |  |  |
|  | *Fos** | NM_005252 | In PCa, c-Fos functions as a proapoptotic agent by repressing the antiapoptotic molecule c-FLIP(L) [39]. | -5.76 | 0.029691 |
|  | *Ets2* | NM_005239 | *Ets2* downregulation in PCa cells inhibited anchorage-dependent and independent growth, cell cycle alterations and induction of apoptotic cell death [40]. | +2.21 | 0.009982 |
| **Adhesion** |  |  |  |  |  |
|  | *Itgb3** | NM_000212 | In PCa cells, functional modulation of these receptors is required for tumor progression within bone tissues [51]. | +4.86 | 0.015519 |
|  | *Itgav** | NM_002210 |  | +8.7 | 0.019915 |
| **Angiogenesis** |  |  |  |  |  |
|  | *Angpt1* | NM_001146 | *Angpt1* promotes blood-vessel stability, enhancing endothelial cell survival [52]. | +12.25 | 0,000213 |
|  | *Vegfa** | NM_003376 | Circulating VEGFA is predictive of biochemical progression in men undergoing radical prostatectomy [43]. | +21.43 | 0.000005 |
| **Invasion and metastasis** |  |  |  |  |  |
|  | *Plau* | NM_002658 | Serum levels of PLAU/uPA correlate with the PCa tumor progression [61]. | +7.63 | 0.000021 |
|  | *Serpine1** | NM_000602 | Elevated tumor *Serpine1* level is associated with a poor prognosis, by maintaining an angiogenic ‘scaffold’ and stabilizing nascent capillary structure [59]. | +9.9 | 0.000601 |
|  | *Mmp9* | NM_004994 | Matrix metallaprotinase-9 plays crucial roles in PCa invasion and metastasis, by degrading extracellular matrix proteins [62]. | +12.62 | 0.004373 |
|  | *Mmp1* | NM_002421 | Overexpression of *Mmp1* in PCa cells increases cell invasion, migration, prostate-tumor growth, and the incidence of lung metastasis [63]. | +19.45 | 0.00162 |

Multiple genes related to cell cycle control and DNA damage repair, apoptosis, signal transduction and gene regulation, cell adhesion, angiogenesis, invasion and metastasis were evaluated for expression levels using the RT2 Profiler PCR Array system. This table lists genes that show significant delta CT (p< 0.05) values and genes with at least a 1.5-fold change in gene expression levels in OPNc-overexpressing cells, relative to empty vector (EV) PC-3 transfected cells. NM indicated gene bank accession numbers. Roles of each gene were drawn from literature references on prostate carcinoma. Positive values indicate up-regulation of individual genes; negative values indicate down-regulation. The data were evaluated by two-tailed Student’s t test. *OPNc - commonly modulated genes in both OvCar-3 and PC-3 carcinoma models.

Cancer Researchcancerres.aacrjournals.org

1. **doi: 10.1158/0008-5472.CAN-07-1310 Cancer Res *October 1, 2007 67; 9425***
